# ClassyFarm and welfare indicators in dairy cattle farming

**DOI:** 10.5455/javar.2026.m1006

**Published:** 2026-03-05

**Authors:** Michela Maria Dimuccio, Edmondo Ceci, Elisabetta Bonerba, Sergio Ghidini, Valentina Terio, Rocco Roma, Gaetano Vitale Celano, Caterina Losacco, Marialaura Corrente, Giancarlo Bozzo

**Affiliations:** 1 Department of Veterinary Medicine, University of Bari Aldo Moro, s.p. per Casamassima, km 3, 70010 Valenzano, Italy; 2 Department of Veterinary Medicine and Animal Science (DIVAS), University of Milan, Via dell’Università 6, 26900 Lodi, Italy; 3 Department of Soil, Plant and Food Sciences (DISSPA), University of Bari Aldo Moro, Bari, Italy; 4 Department of Precision and Regenerative Medicine and Jonian Area, Section of Veterinary Science and Animal Production, University of Bari Aldo Moro, Valenzano, Bari, Italy

**Keywords:** ClassyFarm, cortisol, dairy cattle, *IL-6*, Somatic Cell Count, welfare

## Abstract

**Objectives:**

The study aimed to test whether plasma cortisol, interleukin-6 (*IL-6*), and individual Somatic Cell Count (SCC) can act as reliable proxies for the ClassyFarm risk rank.

**Materials and Methods:**

Three Animal-Based Measures (ABMs) (plasma cortisol, *IL-6*, and SCC) were evaluated and compared with the ClassyFarm risk rank, which is a farm-specific risk score, across three dairy cattle farms (A, B, and C). The ClassyFarm risk rank was 10, 13, and 17 on farms A, B, and C, respectively.

**Results:**

Our findings revealed a strong correlation between plasma cortisol levels and SCC across all herds (*r* > 0.70), particularly in B (*r* = 0.92). The SCC values generally complied with Regulation (EC) No. 853/2004 of the European Commission. However, *IL-6* levels did not show a statistically significant correlation with the other ABMs (*r* < 0.6).

**Conclusions:**

This preliminary study indicates satisfactory agreement between the risk analysis conducted using the ClassyFarm system, as reflected in the ClassyFarm risk rank, and the ABMs investigated. These measures might serve as good real-time proxies for the ClassyFarm risk rank, helping implement any required corrective measures on the farm more promptly.

## 1. Introduction

The farm veterinarian plays a crucial role in ensuring the health, quality, and sustainability of farms, as mandated by articles 3 and 4 of the Decree of the Italian Ministry of Health dated 7 December 2017 [1, 2]. This involves assisting Food Business Operators (FBOs) in drafting and adopting comprehensive farming management plans to ensure high standards of biosecurity and animal welfare, as well as low Antimicrobial Use (AMU) [1, 2, 3, 4, 5, 6]. These plans may include voluntary participation by the farm in the ClassyFarm system, an integrated system devised in 2018 based on instructions from the Italian Ministry of Health to categorize farms according to risk analysis for veterinary public health [6, 7, 8]. ClassyFarm evaluates multiple aspects: (i) biosecurity; (ii) animal welfare; (iii) health and production parameters; (iv) animal nutrition; (v) consumption of antimicrobial drugs; and (vi) lesions detected at the slaughterhouse (e.g., lung score) [9, 10]. This integrated system is currently able to assess various livestock species (swine, cattle, buffalo, sheep, goats, poultry, and rabbits) with specific checklists for each aspect evaluated (e.g., welfare) and subdivided according to the productive aptitude of animals and farming method used [9, 10].

As regards dairy cattle, for example, the ClassyFarm protocol allows monitoring of various subpopulations (heifers, lactating cows, dry cows, transition cows, calves) using separate checklists for closed and open housing [5, 6]. After filling out the different checklists on the field, farm, and official veterinarians upload them to the system (e.g., the “Dairy Cow open housing-Welfare” checklist), and by accessing the Dashboard with the same name as the aspect evaluated, they can download a summary report titled “Data Processing and Summary of Critical Issues Detected in Risk Assessment for Welfare Purposes in Cattle Species”. This document, produced using business intelligence, can be used for various operational needs and will provide a detailed summary of results, including anagraphic farm identification, the name of the veterinarian on duty, and critical control points identified as having insufficient responses in the checklist [11, 12, 13]. ClassyFarm integrates data from official controls, existing information systems, and FBO self-monitoring, in collaboration with farm veterinarians [1, 2, 6, 7]. This integration helps identify risks (i.e., situations found to be non-compliant with ClassyFarm checklists) on each farm and guide interventions to minimize them, enhancing dialogue between FBOs and Competent Authorities and the efficiency of official controls [5, 6, 9].

The ClassyFarm risk rank is determined by algorithms that summarize scores from checklists covering animal welfare, biosecurity, antimicrobial drug use, and other areas. This rank, which reflects each farm’s overall risk, enables constructive comparison with other farms at the local and national levels [14]. According to the ClassyFarm protocol, farms scoring between 1 and 4 must be checked by the competent authority and monitored over time, whereas farms scoring above 4 may be chosen for monitoring [11, 14].

ClassyFarm’s preventive approach aligns with the objectives of epidemiological surveillance, the prevention and control of transmissible diseases, and the combating of antimicrobial resistance (AMR), as set out by the Animal Health Law [3].

Perhaps the most interesting aspect of the ClassyFarm system lies in its use of checklists, including both indirect (Non-Animal-Based Measures or Non-ABMs) and direct (Animal-Based Measures or ABMs) indicators to assess animal welfare. Non-ABMs include Resource and Management-Based Measures (referred to as RBMs and MBMs, respectively) [5]. ABMs highlight existing animal welfare problems, while RBMs and MBMs help identify their causes and future risks [15]. Going more into detail, the ClassyFarm system considers three different risk areas when evaluating animal welfare: (i) area A includes farm management, animal handling, and staff training; (ii) area B encompasses housing facilities; (iii) area C deals with ABMs [7]. The evaluation of areas A and B is carried out by non-ABMs, whilst area C is assessed by ABMs, reflecting the relationship between animals and the surrounding environment [7]. The system’s dual approach to evaluating animal welfare makes this protocol complete and aligned with the concept of Positive Animal Welfare [15]. ABMs are pivotal for assessing the psychophysiological state of animals, in which stress plays a significant role [16, 17]. Stress is an extremely complex phenomenon that, if prolonged, can weaken animals’ immune systems, thus increasing their susceptibility to disease [17].

Cortisol, often referred to as the “stress hormone”, is a glucocorticoid released by the cortical layer of the adrenal glands upon adrenocorticotropic hormone (ACTH) stimulation when the body faces either physical or psychological stressful conditions. Cortisol or its metabolites can be quantified by ELISA using various biological matrices (saliva, blood serum, feces, urine, and milk), and cortisol is a physiological indicator of stress widely used in the field [18, 19, 20], including in ABMs for various species [21]. Recently, methods have been developed for non-invasive measurement of cortisol levels in hair induced by chronic stress [22, 23]. Although the precise mechanism of cortisol incorporation into hair remains unknown, hair cortisol concentration appears to be a potential marker of long-term systemic cortisol excess, as observed in chronic stress [23, 24].

One ABM affecting cortisol levels in the blood is interleukin-6 (*IL-6*). This pleiotropic cytokine, secreted by adrenal cells, influences cortisol release from zona fasciculata (ZF) cells in bovines, rats, and humans [22, 25, 26]. This effect of *IL-6* on adrenocortical cortisol secretion is mediated by the cyclo-oxygenase pathway [25, 26]. Cortisol, on the other hand, increases plasma *IL-6* levels by activating the inflammatory response and the subsequent release of pro-inflammatory cytokines, including *IL-6*, in response to stress [22]. Elevated plasma levels of *IL-6* can be found, therefore, under acute or chronic stress conditions, but also in other cases, such as (i) long-term or short-term inflammatory processes, (ii) osteoarticular trauma and multiple organ injuries, or (iii) intense exercise [22].

Finally, to better understand the connection between animal health and welfare, a 2023 EFSA scientific opinion identified the “welfare consequences” to monitor, including mastitis [27, 28]. Measurement of individual Somatic Cell Count (SCC) is included in ABMs for mastitis control in cows [27]. This routine measurement enables on-farm risks to be identified and, where necessary, corrective actions to be taken after profiling the diseases present on the farm and assessing drug consumption (especially AMU) [29].

Therefore, this preliminary study aimed to test whether the three ABMs (plasma cortisol, *IL-6*, and SCC) can act as reliable proxies for the ClassyFarm risk rank. These parameters were evaluated on the farm and then compared with the ClassyFarm risk rank, which is a farm-specific risk score, across three dairy cattle farms (A, B, C).

## 2. Materials and Methods

### 2.1. Ethical approval

The experimental procedures were approved by the ethical committee of the Department of Veterinary Medicine at the University of Bari “Aldo Moro”, Italy (Approval Number n. 20/23, Prot. N. 2645-III/13of 20 June 2023).

### 2.2. Sampling

The result of a collaboration between the Food Safety Section of the Department of Veterinary Medicine (DiMeV) at the University of Bari – Aldo Moro (Italy) and the Veterinary Service of Livestock Hygiene and Livestock Production of the Apulia Region, this study was carried out in May and June 2024.

The study involved three dairy cattle farms in the province of Brindisi participating in the ClassyFarm system, hereinafter referred to as farms A, B, and C, which respectively obtained ClassyFarm risk ranks of 10, 13, and 17 in 2023 (the latest data available from the system).

The number of animals was equal to 655 on farm A, with 277 lactating cows, 383 on farm B, with 116 lactating cows, and 278 on farm C, with 148 lactating cows, respectively. The study involved sampling 15% of lactating cows per farm (in their 3rd or 4th lactation) that received the same commercial feed ad libitum and were managed in a loose-housing system. The total number of sampled animals was 80, divided into 41, 17, and 22 lactating cows from farms A, B, and C, respectively.

The sample selection was conditioned by the availability of Apulian livestock farms participating in the ClassyFarm system for at least 2 years (so as to already have a ClassyFarm risk rank published) and having different ClassyFarm risk ranks during the scheduled sampling period. In this preliminary study, the lactating cow’s percentage resulted from the number of animals made available by operators for experimental testing and from the need to obtain a sample as homogenous as possible.

Blood samples were collected from the tail vein by professionally trained personnel (veterinarians from the local Veterinary Animal Health Service) at the same time (9:00 a.m.) on each farm on the same day of the week for three weeks to exclude a circadian variation of cortisol levels. The same personnel were involved in both collecting samples and comparing them with the animal welfare data for each farm available in the ClassyFarm system.

The blood samples were collected in 13 × 75 mm vacuum test tubes (BD Vacutainer^®^ EDTA Tubes) containing ethylenediaminetetraacetic acid (EDTA) and stored on ice at 0°C for no longer than 60 min, thus avoiding freezing, before being sent to the DiMeV laboratories. Samples were processed to measure cortisol and *IL-6* levels using a Bovine Cortisol ELISA Kit and a Bovine *IL-6* ELISA Kit, respectively. Plasma cortisol and *IL-6* levels were measured using the protocol outlined by Ceci et al. [18]. The ELISA immunoassays for cortisol and *IL-6* were performed according to the manufacturer’s guidelines (Bovine Cortisol ELISA; My-Bio-Source, San Diego, CA, USA, and Bovine *IL-6* ELISA; My-Bio-Source, San Diego, CA, USA, respectively). Prior to reconstitution, all reagents were kept at room temperature (25–28°C) for 30–40 min. The enzyme conjugate required for the analyses was stored at –20°C until use. Both ELISA tests were performed using a DYNEX DSX^®^ fully automated four-plate ELISA processing system. DSX^®^ is a proven, automated open system that performs multiple assays per plate simultaneously, delivering optimized efficiency and speed. It uses a perfectly synchronized stem to prevent plate drift, ensuring consistent results across its four plate incubators.

Additionally, individual milk samples were collected, stored at 4°C, and analyzed within 24 h in compliance with ISO 13366-2/IDF 148-2:2006. SCC, expressed as cells/ml was quantified using a Fossomatic FC (Foss Analytical A/S, Foss Allé 1, DK-3400 Hillerød, Denmark) operated as a fluorescence microscope. The method employs ethidium bromide, a dye that penetrates cells and binds to nuclear DNA, forming a fluorescent complex. Each marked cell generates an electrical pulse, detected and recorded by an optical system at a predetermined wavelength [30].

### 2.3. Statistical analysis

The mean values of the three different ABMs across the three farms were compared using one-way analysis of variance (ANOVA). Data analysis was conducted with IBM SPSS Statistics for Windows, Version 21.0 (IBM Corp., Armonk, NY, USA). A *p*-value of less than 0.05 was considered statistically significant. Correlation among the three parameters was assessed using Pearson’s correlation coefficient.

## 3. Results and Discussion

**Figure 1.**
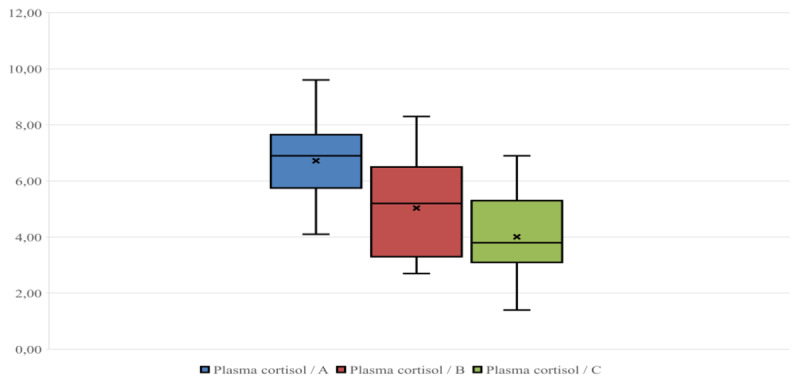
Plasma cortisol (ng/ml) distribution in the three groups considered (A, B, C). The line inside each box indicates the median, and the “×” indicates the mean.

**Figure 2.**
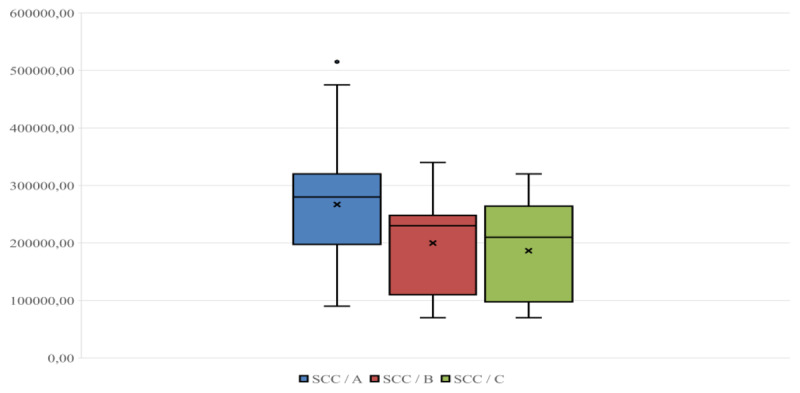
SCC (cells/ml) distribution in the three groups considered (A, B, C). The line inside each box indicates the median, and the “×” indicates the mean.

**Figure 3.**
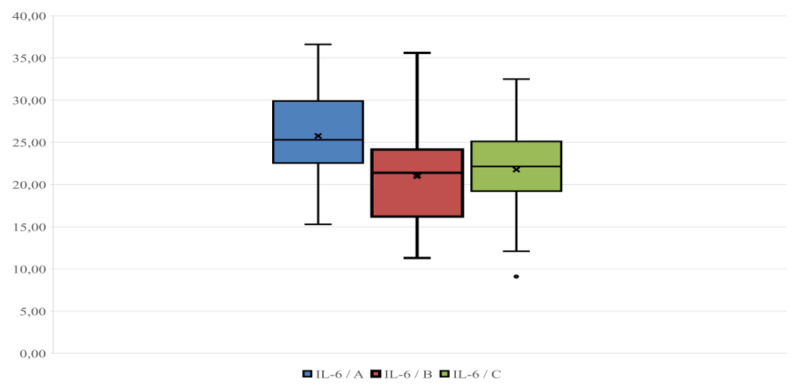
*IL-6* (ng/ml) distribution in the three groups considered (A, B, C). The line inside each box indicates the median, and the “×” indicates the mean.

Plasma cortisol levels ranged between 4.1 and 9.6 ng/ml; 2.7 and 8.3 ng/ml; and 1.40 and 6.90 ng/ml on farms A, B, and C (Figure 1), respectively; the average respective cortisol values were 6.71 ± 1.25; 5.03 ± 1.70, and 4.01 ± 1.54 ng/ml. SCC levels ranged between 90,000 and 475,000 cells/ml, 70,000 and 340,000 cells/ml, and 70,000 and 320,000 cells/ml on farms A, B, and C, respectively (Figure 2); the respective average SCC values were 266,878 ± 102,940, 199,764 ± 82,047, and 186,500 ± 87,457 cells/ml. Finally, plasma levels of *IL-6* ranged between 15.3 and 36.6 ng/ml, 13.6 and 35.6 ng/ml, and 9.1 and 28.8 ng/ml on farms A, B, and C, respectively (Figure 3); the respective average levels of *IL-6* were 25.65 ± 5.55, 21.02 ± 6.40, and 21.66 ± 5.47 ng/ml.

As shown by the box plots (Figures 1, 2, 3), the small distance between the mean and median makes the distributions of observations across the different parameters close to normal [31]. This is evident in all three parameters considered in the study, particularly for plasma cortisol and *IL-6* (Figures 1, 3). Within each Figure, the height of each box, known as the interquartile range, covers 50% of the distribution and indicates the dispersion of the variable relative to the median across the three farms. Furthermore, the line inside each box indicates the median, and the “×” indicates the mean.

All the plasma cortisol values observed fell within baseline cortisol levels physiologically observed in lactating dairy cows [32], thus indicating the absence of stressful stimuli on farms A, B, and C shortly before blood samples collection (acute stress). The HPA axis activity in lactating dairy cows, as shown by Beerda et al. [33] and Munksgaard et al. [34], differs from that observed in growing animals [33, 34]. This different adrenal reactivity in dairy cows is explained by considering the negative energy balance during post-partum and lactation and by the role of cortisol in mobilizing body fat reserves [22, 32, 35].

In addition, awakening and milking time, which may vary from farm to farm depending on the operator’s routine and the farm’s geographic location along the milk-collecting route, should be considered for adequate sampling.

To obtain a blood sample unaffected by circadian cortisol rhythms or morning milking, the sampling time should be defined for each farm in collaboration with its FBO and farm veterinarian.

It is, in any case, important to collect samples at the same time on each farm during subsequent samplings, scheduling with the highest possible frequency, the lower the ClassyFarm risk rank is. However, as reported by Gross et al. [32], it would be wise to select cows that have exceeded 100 days of lactation and wait 120 min after morning milking before collecting an ideal blood sample for cortisol testing. Regarding the sample’s transport to the lab, it should be done as soon as possible (ideally within 3 h) to avoid potential alterations in the *IL-6* assay [36].

The SCC represents the total count of immune cells in milk and is a well-known indicator of udder health. Primarily, it is used to detect intra-mammary infections, a major cause of bovine mastitis [37, 38, 39]. Apart from four animals on farm A, SCC levels were found to comply with the requirements (SCC ≤ 400,000) set by Regulation (EC) No 853/2004 (section IX chapter III) [40]. These results, while meeting the legal limit, indicate that the cows’ immune system was involved in the mammary region, even if the inflammatory process was not yet clinically evident [39, 41]. Bovine mastitis is frequently subclinical or chronic, suspected when SCC values are > 100,000 cells/ml, along with changes in other parameters used to assess dairy cow welfare and health (i.e., plasma cortisol and *IL-6*) [39, 41]. In this regard, consideration needs to be given to the fact that the SCC values found were highly correlated with plasma cortisol levels in the three herds (*r* > 0.70), and particularly in herd B (*r* = 0.92) (data not shown). SCC levels exceeding 100,000 cells/ml should be closely monitored by FBOs and farm veterinarians as an early indicator of udder health changes. Whenever possible, the sources of gradual increases in SCC levels (stress factor and/or infectious agent) should be identified and addressed. This preventive and proactive approach, in line with the requirements of Regulation (EU) No 2016/429 to prevent and control transmissible animal diseases, enables the reduction of disease onset and spread on-farm and, consequently, the AMU [3, 6]. In turn, a lower AMU decreases the costs of veterinary care and of the safe disposal of milk from treated animals, with economic benefits for the FBO [16]. Monitoring SCC variations and collecting data on AMU help limit the emergence of new resistant strains while preserving the efficacy of veterinary and human antibiotics through optimized use [16, 42, 43, 44].

The *IL-6* values were not highly correlated with the other two ABMs assessed in this study (*r* < 0.6) on farms B and C, whereas a greater correlation was observed on farm A (data not shown). This finding does not fully align with the consulted literature [45, 46, 47]. Some previous studies showed that this cytokine serves as a marker of inflammation in subclinical mastitis in dairy cows, and that this intra-adrenal factor is involved in the regulation of the stress response through adrenal steroid secretion [45, 46, 48].

To compare the three farms simultaneously, an ANOVA was performed, which showed statistically significant differences across all three ABMs (Table 1). Comparing the farms in pairs (Tables 2, 3, 4), the largest variation in ABMs was found between farm A and the other two (B, C). There were no significant differences for the three ABMs between farms B and C. In agreement with Reyes et al. [49], one explanation for this could be the higher number of animals on farm A (*n* = 655) compared with farms B (*n* = 383) and C (*n* = 278), as a greater number of animals/interactions would seem to influence their psycho-physical welfare. Notably, farm A also had the lowest ClassyFarm risk rank [49, 50].

**Table 1.** A One-Way ANOVA was performed to compare the mean values of the three ABMs across the three farms simultaneously. *p*-values are considered significant at *p* < 0.05.

		**Sum of Squares**	**Df**	**Mean of Square**	**F**	***p*-value**	**F critical value**
Plasma cortisol (ng/ml)	Between groups	112.43	2	56.21	27.16	.001	3.11
	Within groups	159.37	77	2.07			
	Total	271.81	79				
SCC (cells/ml)	Between groups	112909038432.58	2	56454519216.28	6.28	.001	3.11
	Within groups	692200949067.43	77	8989622715.16			
	Total	805109987500.00	79				
*IL-6* (ng/ml)	Between groups	376.24	2	188.12	5.86	.001	3.11
	Within groups	2468.57	77	32.05			
	Total	2844.81	79				

**Table 2.** A One-Way ANOVA was performed to compare pairs of farms for plasma cortisol (ng/ml). *p*-values are considered significant at *p* < 0.05.

		**Sum of Squares**	**Df**	**Mean of Square**	**F**	***p*-value**	**F critical value**
Plasma cortisol A *vs*. B	Between groups	34.08	1	34.08	17.44	.001	4.01
	Within groups	109.40	56	1.95			
	Total	143.49	57				
Plasma cortisol B *vs*. C	Between groups	10.08	1	10.08	3.86	0.06	4.10
	Within groups	96.49	37	2.60			
	Total	106.58	38				
Plasma cortisol A *vs*. C	Between groups	105.14	1	105.14	56.83	.001	3.99
	Within groups	112.85	61	1.855			
	Total	218.00	62				

**Table 3.** A One-Way ANOVA was performed to compare pairs of farms regarding SCC (cells/ml). *p*-values are considered significant at *p* < 0.05.

		**Sum of Squares**	**Df**	**Mean of Square**	**F**	***p*-value**	**F critical value**
SCC A *vs*. B	Between groups	54128068173.94	1	54128068174	5.70	.001	4.01
	Within groups	531577449067.43	56	9492454448			
	Total	585705517241.37	57				
SCC B *vs*. C	Between groups	1687338612	1	1687338612	0.23	0.63	4.10
	Within groups	268332558823.52	37	7252231320			
	Total	270019897435.89	38				
SCC A *vs*. C	Between groups	92499824042	1	92499824042	9.65	.001	3.99
	Within groups	584491890243.90	61	9581834266			
	Total	676991714285.71	62				

**Table 4.** A One-Way ANOVA was performed to compare the farms in pairs for *IL-6* (ng/ml). *p*-values are considered significant at *p* < 0.05.

		**Sum of Squares**	**Df**	**Mean of Square**	**F**	***p*-value**	**F critical value**
*IL-6* A *vs*. B	Between groups	268.87	1	266.87	8.01	.001	4.01
	Within groups	1878.91	56	33.55			
	Total	2147.78	57				
*IL-6* B *vs*. C	Between groups	5.38	1	5.38	0.15	0.69	4.10
	Within groups	1247.05	37	33.70			
	Total	1252.43	38				
*IL-6* A *vs*. C	Between groups	226.90	1	226.90	7.64	.001	3.99
	Within groups	1811.18	61	29.69			
	Total	2038.08	62				

As reported in Table 2, the F-value for plasma cortisol was 3.86, while the *p*-value was 0.06; whereas the F-value for SSC was just 0.2, while the *p*-value was 0.63 (Table 3). Lastly, the F-value for *IL-6* was 0.15, while the *p*-value was 0.69 (Table 4).

The absence of statistically significant differences between farms B and C, despite having different ClassyFarm risk ranks (13 *vs*.17, respectively) might suggest that in ClassyFarm system shows no significant differences in terms of animal welfare, when the risk rank exceeds 13, far above the cut-off risk rank of 4, that in ClassyFarm indicates a need for official controls to be carried out on-farm [12]. Moreover, higher scores indicate better farm management, even when there is room for improvement across various areas (welfare, biosecurity, etc.), as evidenced by checklists and comparisons of farms [12]. Animal welfare assessment on farms, as established by the ClassyFarm system, may seem complex and difficult to correlate with ABMs, which are not yet included in this software, though their validity is well established [18, 22]. Thus, this preliminary study showed promising agreement between the risk analysis conducted using the ClassyFarm system and the assessed ABMs (plasma cortisol, SCC, and *IL-6*); therefore, according to our findings, a high ClassyFarm risk rank will be associated with lower correlations among ABMs.

## 4. Conclusions

This preliminary study showed that the three investigated ABMs might serve as a valuable real-time proxy for the ClassyFarm risk rank, issued by the health authorities only every 12 months, helping to implement corrective measures on the farm in a timely manner. Enhancing animal welfare and biosecurity on farms is crucial for improving animal health, reducing reliance on drug treatments, and minimizing antimicrobial use. This approach will also positively affect the ClassyFarm risk rank, increasing the FBO’s chances of accessing Common Agricultural Policy (CAP) 2023–2027 funding. Additionally, plasma cortisol, SCC, and *IL-6* levels should be monitored with a frequency that reflects the ClassyFarm risk rank (the lower the score, the higher the frequency of controls). However, these findings require further validation using larger sample sizes across both livestock farms and the animals involved.

## Data Availability

The data presented in this study are available from the corresponding author upon reasonable request.

## List of abbreviations

ABMs, animal-based measures; SCC, Somatic cell count; *IL-6*, interleukin-6; FBOs, food business operators; AMU, antimicrobial use; non-ABMs, non-animal-based measures; RBMs and MBMs, resource and management-based measures; DiMeV, Department of Veterinary Medicine; ANOVA, analysis of variance; CAP, common agricultural policy; ng/ml, nanograms/milliliter; cells/ml, cells/milliliter; min, minutes; h, hours.
